# Remarkable Case of Uterine Hemorrhage, Terminating Fatally

**Published:** 1848-06

**Authors:** F. L. Sewall

**Affiliations:** Jackson, Clark county, Ala.


*Remarkable Case of Uterine Hemorrhage, terminating fatally.* By F. L. Sewall, M. D., of Jackson, Clark county, Ala.

Dr. R. M. Huston :

Sir,—If the subjoined detail of a case which lately fell under my observation, is of sufficient interest to merit a place in your journal, it is at your service.

On the 22d ult. a negro woman, slave of Mrs. H. near this village, aged 42, stout, strong, and in her usual health, after eating a full dinner, was engaged in light work some one hundred paces from the house ; she was observed suddenly to stop and call for assistance. Mrs. H. started to her—the woman exclaimed make haste ! Before Mrs. H. could reach her, she fell and never spoke again, but died within twenty minutes, the ground around her being covered with blood, apparently from uterine hemorrhage.

Early in life this woman had borne a child; subsequently she had been barren—suffering under dysmenorrhea. These are the facts preceding the death, as I learned from Mrs. H., a lady of good sense. I ought, however, to say more explicitly, that *nothing* but blood passed from the woman. With some difficulty I obtained permission to examine the body, assisted by my friend Dr. Baker. Externally the body presented nothing unusual. The uterus and ovaria were removed. The uterus was found hard and firm, and perhaps a little over usual size. Opening it, the walls were found to be eight or ten lines in thickness, of a uniform yellowish white colour. In the fundus of the uterus and imbedded in its substance, was a fibrous tumour three-fourths of an inch in diameter.

The cavity was filled with blood partially coagulated, but we found no ulceration, no abrasion, or any other unusual appearance; the cervix uteri about three-fourths of an inch in length, and with a diameter nearly or quite equal to its length, presented a white, shining and cartilaginous appearance, and the os uteri scarcely large enough to admit a *small* silver probe. The ovaria presented a shrunken appearance, and each one contained several small vesicles or cysts filled with dark blood.

I examined the place where she fell; there was neither stick nor stone; she could not then have sustained any external violence.

I will make no comments on the case, but say simply in conclusion, that I am not satisfied as to the cause or source of such a rapid loss of blood. I should be gratified to learn your opinion on the subject.

[The case narrated above seems to have been one of fatal syncope, arising from a sudden loss of blood; and, as the heart and great blood vessels were not examined, it is possible that lesions existed in some part of the circulatory apparatus, predisposing the individual to such a termination. As the cavity of the uterus "was filled with blood partially coagulated," and no ulceration or abrasion existed, the loss of blood was doubtless owing to a rapid exhalation from the lining membrane, such as frequently occurs from the lungs, stomach and intestines, nasal passages, &c. The fibrous tumour found in the fundus of the uterus, it is probable, had no agency in causing the hemorrhage. Such formations are constantly found in the organ, of considerable size, without causing very serious disturbance.—Ed.]

